# The Catoptric Test for Cataract

**Published:** 1851-05

**Authors:** James Bolton


ARTICLE VII.

The Catoptric Test for Cataract.—A paper read before the Medical Society of Virginia, by James Bolton, M.D.

The diagnosis of cataract is often extremely puzzling. In amaurosis, an opacity is often observed behind the pupil, which resembles an opaque crystalline lens.

When we consider the extreme value of the affected organ, and the totally diverse methods of treatment applicable to the two diseases thus liable to be confounded, we are prepared to place a high estimate upon any means which may enable us to form an accurate diagnosis.

If the opacity be produced by inflammation of the vitreous humor and retina, the puncture and laceration of these tissues, required by the operation for cataract, would probably increase the existing inflammation, and render the case hopeless.

When the anterior surface of the lens is opaque, owing to loss of transparency of the anterior surface of its capsule, or of its substance, or of both combined, the diagnosis is comparatively easy.

There are three principal points which distinguish the opacity from an affection of the vitreous humor. 1st, Its position. It appears nearly in contact with the iris at its pupillary margin. 2d, Its form being convex. 3d, It does not disappear on taking an oblique view of it. Opacity of the vitreous humor is more deeply seated; its form is concave; and it can be seen only by a direct front view.

When the posterior surface of the lenticular capsule has lost its transparency, the opacity corresponds, both in form and position, with opacity of the vitreous humor; and the resemblance will be still more perplexing if the cataract be in the nascent state.

In such cases, the highly philosophical, delicate and accurate test of Sanson, called also the Catoptric Test, is of inestimable value.

The following is a description of it:

If a clear flame of a candle be held before a healthy eye, there will be seen upon the surface of the cornea, a bright, well-defined image of the flame, erect, and very much diminished in size. On looking deeply into the eye, through the pupil, there will be seen a faint, bluish image, with an outline not well defined, erect, and larger than the first. Between these two may be seen a third image, bright like the first, very clearly defined, much smaller than the other two, and inverted. There are then two erect images, and an inverted one between them. On moving the candle across the eye, the erect images will be observed to follow it, while the inverted one moves in the opposite direction. The outer one traverses the entire cornea, and the other two pass no farther than the margin of the pupil.

These phenomena are explained by the fact, that when a ray of light penetrates a healthy eye, it encounters three reflecting surfaces: first, the convex cornea; second, the convex anterior portion of the lenticular capsule; third, the concave posterior portion of the same membrane. The two first and second reflect upright images; the third, being a concave mirror, reflects an inverted one, which falls between the other two, in consequence of that being the position of its focus.

These images furnish us with very important indications.

Obliteration of all three images indicates opacity of the cornea, without giving any information as to the condition of the interior structure of the eye. Obliteration of the inverted image only, indicates opacity of the posterior surface of the lens, with transparency of its anterior surface, and of the cornea. Obliteration of the posterior erect image indicates opacity of the anterior surface of the lens, and must necessarily be accompanied by obliteration of the inverted one.

The most important of these images is the inverted one. If that can be made out distinctly, there is no cataract; for the posterior surface of the lens must be clear, in order to reflect the rays of light, and the body and anterior surface of the lens must be clear, in order to transmit these rays.

In amaurosis, therefore, all three images are visible; in cataract, when fully formed, only one, the corneal image can be seen.

But this method of diagnosis is available for the detection of not only matured cataract, but such is its mathematical precision, that the least departure from a healthy condition of these reflecting surfaces is invariably indicated by a corresponding departure from the normal appearance of the images which they present. It not only detects the existence of cataract, but indicates its state of progression from incipiency to maturity.

In the whole range of medical science, I know of no method of detecting disease more beautifully illustrative of physical science, and none which may be relied on with more implicit confidence.

The following case, although unfortunate in its termination, exemplifies these remarks in an interesting manner:

J. F., bookbinder, about 50 years of age, intemperate, has been blind in the left eye for several years, during which it has been subject to attacts of inflammation.

There is sufficient vision to distinguish night from day.

Behind the pupil, and very deeply situated is an opacity of a brownish muddy color. Its form is indistinct. The sight of the other eye is defective, but all the images are visible.

*Diagnosis.*—Opacity of the posterior part of the capsule of the right eye, with probably some degree of amaurosis, which already exists in the left eye.

*Operation.*—Anæsthesia was produced by chloroform. An incision was made in the superior margin of the cornea, through which the lens was extracted. On the posterior surface of the capsule was an opacity, darker at its centre and shaded off to an undefined edge. The diagnosis was therefore accurately confirmed. The patient was confined to bed, cold water was applied locally, and a strictly antiphlogistic regimen enforced. On the fourth day the patient accidentally used the eye for a moment, and distinctly saw some small object. In a day or two more the eye was suddenly attacked by violent inflammation and pain, which were relieved by active antiphlogistic measures, but the eye became hopelessly amaurotic.—*Stethoscope.*

